# Idiosyncratic liver injury induced by bolus combination treatment with emodin and 2,3,5,4′-tetrahydroxystilbene-2-*O*-*β*-D-glucopyranoside in rats

**DOI:** 10.3389/fphar.2022.1017741

**Published:** 2022-09-26

**Authors:** Dan Li, Yuanfeng Lyu, Qianbo Song, Yuen Sze Lai, Zhong Zuo

**Affiliations:** School of Pharmacy, Faculty of Medicine, The Chinese University of Hong Kong, Hong Kong, China

**Keywords:** polygoni multiflori radix, idiosyncratic liver injury, emodin, bile acid homeostasis, apoptosis, 2,3,5,4′-tetrahydroxystilbene-2-O-β-D-glucoside

## Abstract

Polygoni Multiflori Radix (PMR) is a commonly used traditional Chinese medicine in clinical practice, while adverse effects of hepatotoxicity related to PMR have been frequently reported. The clinical case reports indicated that PMR hepatotoxicity could occur under both overdose medication/long-term exposure and low doses with short-duration (idiosyncratic) conditions. The combination treatment with emodin and 2,3,5,4′-tetrahydroxystilbene-2-*O*-*β*-D-glucopyranoside (TSG), two major PMR components, was reported to contribute to PMR hepatotoxicity after long-term treatment. However, the role of the combination treatment of these two components in PMR-induced idiosyncratic liver injury has not been clearly clarified. In this study, the LPS-mediated inflammatory stress model rats were adopted to explore the idiosyncratic liver injury induced by the bolus combination treatment with emodin and TSG. After a bolus oral administration with TSG (165 mg/kg), emodin (5 mg/kg) or their combination in both normal and LPS-mediated inflammatory stress model rats, the systemic/hepatic concentrations of emodin, emodin glucuronides and bile acids were determined; the hepatotoxicity assessments were conducted *via* monitoring histopathological changes and liver injury biomarkers (ALT and AST). Moreover, the protein expressions of bile acid homeostasis- and apoptosis-related proteins were examined. No liver damage was observed in the normal rats after a bolus dose with the individual or combination treatment, while the bolus combination treatment with emodin and TSG induced liver injury in the LPS-mediated inflammatory stress model rats, evidenced by the elevated plasma levels of alanine aminotransferase (∼66%) and aspartate aminotransferase (∼72%) accompanied by severe inflammatory cell infiltration and apoptotic hepatocytes in liver tissue. Moreover, such combination treatment at a bolus dose in the LPS-mediated inflammatory stress model rats could significantly elevate the hepatic TBA levels by about 45% *via* up-regulating the hepatic protein expression levels of bile acid synthesis enzymes and inhibiting that of bile acid efflux transporters and the expression levels of apoptosis-related proteins. Our study for the first time proved the major contribution of the combination treatment with emodin and TSG in PMR-induced idiosyncratic liver injury.

## 1 Introduction

Polygoni Multiflori Radix (PMR) is originated from the dried roots of *Polygonum multiflorum* Thunb. and it has been used as traditional Chinese medicine or health supplement for centuries. However, the constantly reported liver injury adverse effects induced by PMR or the PMR-containing proprietary Chinese medicines products have aroused great concern around the world (But et al., 1996; Park et al., 2001; Cárdenas et al., 2006; Laird et al., 2008; Cho et al., 2009; Jung et al., 2011; Dong et al., 2014). Among the reported liver injury cases of PMR, it was noted that some cases even resulted from orally administered PMR at a low dose for a short duration (e.g., 3 g/person/day for 1 day) (Lei et al., 2015). In addition, preclinical studies have reported that PMR extract could lead to liver injury in inflammation model rats triggered by the bacterial lipopolysaccharides (LPS) (Gao et al., 2016b; Li et al., 2016; Tu et al., 2019b). More recently, Li et al. suggested that the allele human leukocyte antigen (HLA)-B*35:01 was highly related to PMR-induced liver injury with a higher incidence rate (37.5%, 3 of 8 patients) for the patients carried with HLA-B*35:01 in comparison to the noncarriers (4.7%, 3 out of 64 patients) (Li et al., 2019). Therefore, PMR-induced liver injury was suggested to possess the properties of idiosyncratic drug-induced liver injury (DILI).

Unlike the intrinsic DILI, which is typically dose-dependent occurring after reaching the threshold dose or exposure level, the idiosyncratic DILI occurs unpredictably (Cortes et al., 2020). It is usually independent of dose level, administration route, duration of medication exposure and its known pharmacological actions (Cortes et al., 2020). The idiosyncratic DILI is considered to be the result of complicated interactions among several critical factors, including toxicological drug properties, host-and environmental-related factors (Chen et al., 2015). To clarify the major components contributing to the PMR-induced idiosyncratic liver injury, hepatotoxic evaluations of pure PMR components on the LPS-mediated inflammatory stress model rats were conducted and found that emodin, *cis*-2,3,5,4′-tetrahydroxystilbene-2-*O*-*β*-D-glucopyranoside (*cis*-TSG), or the combination treatment with emodin-8-*O*-*β*-D-glucopyranoside (EMG) and *trans*-TSG could all lead to liver injury at bolus dose only in the LPS-mediated inflammatory stress model rats with no impact on the normal rats (Tu et al., 2015; Meng et al., 2017; Zhang et al., 2020). Since *trans*-TSG was reported to be the dominant form in the PMR due to its higher stability than the *cis*-form (Gao et al., 2016a) and showed no hepatotoxicity in the LPS-mediated inflammatory stress model rats (Meng et al., 2017; Zhang et al., 2020), *cis or trans*-TSG may not be the major reason behind the PMR-induced idiosyncratic liver injury. In addition, as demonstrated in previous studies (Li et al., 2020; Li et al., 2021), the systemic or hepatic exposure of EMG was much lower than that of emodin despite its higher content (0.029–0.365% w/w) in PMR than that of emodin (0.0064–0.082% w/w) (Zhang et al., 2018; Li et al., 2021; Cheng et al., 2022) and EMG could be hydrolysed to emodin *via* intestinal microflora (Ullah et al., 2018), suggesting the higher potency of emodin than EMG in PMR-induced liver injury. Therefore, among the above-mentioned potential hepatotoxic components, emodin is considered to be the major component contributing to PMR-induced idiosyncratic liver injury. Moreover, our recent study demonstrated that emodin possesses the strongest hepatotoxic effect among the tested PMR major components and the presence of TSG could further enhance its hepatotoxicity in both the sandwich-cultured rat hepatocytes (SCRH) and the normal rats after consecutive treatment for 21 days *via* increasing the emodin exposure and disrupting the bile acid homeostasis (Li et al., 2022). However, whether the combined treatment with emodin and TSG at a bolus dose could induce idiosyncratic liver injury and the underlying mechanisms remain unknown. Therefore, the LPS-mediated inflammatory stress rat model was adopted in the current study to further investigate the bile acid homeostasis-based hepatotoxic effect after the bolus dose treatment with emodin, TSG or their combination.

## 2 Materials and methods

### 2.1 Chemicals and reagents

Emodin (purity > 98%) and TSG (purity > 98%) were obtained from Chengdu Must Bio-Technology Co., Ltd. Bicinchoninic acid (BCA) protein assay kit, 30% bovine serum albumin (BSA), carboxymethylcellulose sodium (CMC-Na) with molecular weight at 250,000, lipopolysaccharide (LPS), RIPA lysis buffer, phosphate-buffered saline (PBS) tablet, protease inhibitor cocktail, as well as the bile acids standards were purchased from Sigma-Aldrich (St. Louis, MO, United States). Alanine aminotransferase (ALT), aspartate aminotransferase (AST), interleukin 1 beta (IL-1β), IL-6, tumor necrosis factor-alpha (TNF-α) and interferon-gamma (IFN-γ) ELISA kits, as well as the terminal deoxynucleotidyl transferase (TdT) dUTP nick-end labeling (TUNEL) assay kit were purchased from Abcam (Cambridge, MA, United States). The deoxyribonuclease I (DNase I) was supplied by Worthington Biochemical Corporation (Lakewood, NJ, United States). Primary antibodies for cholesterol 7α-hydroxylase (CYP7A1), sterol 27-hydroxylase (CYP27A1), bile salt export pump (BSEP), BAX, BCL-2 were supplied by Santa Cruz Biotechnology (Dallas, TX, United States), multidrug resistance-associated proteins 2 (MRP2) and MRP3 were from Thermo Fisher Scientific (Cleveland, OH, United States), and that of Cleaved CASPASE-3, Cleaved CASPASE-9, MRP4, β-ACTIN and all secondary antibodies were purchased from Abcam (Cambridge, MA, United States). Acetonitrile (ACN) and methanol (MeOH) in High-performance liquid chromatography (HPLC) grades were purchased from RCI Labscan Limited (Bangkok, Thailand). Distilled deionized water was produced by the Millipore water purification system (Milford, MA, United States). All other chemicals and reagents are commercially available.

### 2.2 Animals

Adult SD rats (male, weighing 180–200 g) were purchased from the Laboratory Animal Services Centre, the Chinese University of Hong Kong, Hong Kong SAR, P.R. China. All rats were housed in the temperature (25 ± 2°C) and humidity (50 ± 5%) controlled conditions under a 12/12 h light-dark cycle and free access to food and water. All the animal study was approved by the Animal Experimentation Ethics Committee of the Chinese University of Hong Kong (Reference No. 20–178-MIS, Approved on 5 November 2020).

### 2.3 Animal treatment

Emodin or TSG was suspended in 0.5% CMC-Na to obtain a final concentration of 1 mg/ml and 33 mg/ml, respectively. Forty-eight SD rats were divided into eight groups randomly with six rats per group in four normal rat groups (NG1 to NG4) and four LPS-mediated inflammatory stress model rat groups (LPSG1 to LPSG4). Rats in NG1/LPSG1 received bolus oral administration of 0.5% CMC-Na as control, while those in NG2/LPSG2, NG3/LPSG3, and NG4/LPSG4 were orally administered with 165 mg/kg TSG suspension, 5 mg/kg emodin suspension and 165 mg/kg TSG together with 5 mg/kg emodin, respectively. Three hours after oral administration, rats in NG1 to NG4 were injected with normal saline and those in LPSG1 to LPSG4 were injected with 2.8 mg/kg LPS (Li et al., 2015) dissolved in normal saline *via* the tail vein. Rats from all groups were sacrificed at 10 hours post the oral treatments *via* overdose of xylazine (10 mg/kg) and ketamine (100 mg/kg) followed by collecting blood through the iliac artery and livers. The plasma was obtained *via* centrifuging the collected blood samples at 8,000 g for 3 min to determine inflammatory cytokines, liver injury biomarkers, and emodin and bile acids concentrations. While part of the livers was fixed in the 4% formalin for the histological examinations. To determine the hepatic concentrations of emodin and bile acids or examine the protein expression levels of interested molecules, the collected liver was homogenized with water (1:2, w/v) or RIPA lysis buffer containing protease inhibitors (1:10, w/v) to obtain liver homogenate, respectively.

### 2.4 Sample analyses

#### 2.4.1 Plasma biochemical and inflammatory cytokines analyses

To evaluate the inflammatory responses stimulated by the LPS, the plasma levels of IL-1β, IL-6, IFN-γ, and TNF-α were determined *via* the corresponding ELISA kits according to the manufacturers’ instructions. In the meantime, the plasma levels of ALT and AST, the biochemical markers for hepatic damage, were also determined *via* the commercially available ELISA kits.

#### 2.4.2 Liver histopathological examination and apoptosis evaluation

The liver tissues were fixed in 4% neutral buffered formalin, then embedded in paraffin, sectioned to a thickness of 5 µm followed by dewaxing and staining with hematoxylin and eosin (H&E) sealing with neutral resin. Meanwhile, the paraffin slices of the livers were conducted the TUNEL staining according to the manufacturer’s instructions of the TUNEL assay kit. In brief, after rehydration, the specimens were permeabilized and quenched by treating with proteinase K and 3% H_2_O_2_, respectively, followed by labeling the TdT with the help of TdT enzymes. The labeled specimens were incubated with the blocking buffer followed by staining with the DAB solution. To prepare a positive control for the TUNEL staining assay, the liver sample was pre-treated with proteinase K prior to incubation with 1 μg/μL DNase I for 20 min and subsequent labeling TdT with TdT enzyme. To prepare the negative control, TdT enzyme was replaced by water during the above-mentioned TdT labeling process. The prepared slices were visualized under the light microscope (Eclipse Ti-E, Nikon, Japan) equipped with a digital video camera (DS-Qi2, Nikon, Japan) and NIS-Elements Imaging software for histopathology evaluation of liver damage and apoptosis. The apoptotic hepatocytes in the TUNEL assay images for each sample were scored semi-quantitatively by ImageJ software *via* averaging the gray value intensity of the identified apoptotic hepatocytes/field at ×100 magnification. The obtained scores for samples from normal rats treated with emodin, TSG or their combination (NG2, NG3, NG4) as well as all the LPS treatment groups (LPSG1, LPSG2, LPSG3 LPSG4) were normalized with that of the normal vehicle control group (NG1) prior to their comparison.

#### 2.4.3 Determination of emodin and its glucuronides in rat plasma and liver

Plasma and hepatic concentrations of emodin and its glucuronides from rats receiving emodin (NG3, NG4, LPSG3, and LPSG4) were analysed with our developed HPLC-MS/MS method described in the previous report (Li et al., 2021). In brief, the internal standard (IS) solution (daidzein, 10 μL, 5 μg/ml) was added to 100 μL liver homogenate or plasma followed by the addition of 50 μL acetate buffer (0.5M, pH 5) and 50 μL ascorbic acid (100 mg/ml) and vortexing for 30 s. After acidifying the resulted mixture with 50 μL 0.1% formic acid and extracting with 1 ml ethyl acetate twice, the pooled organic layer was obtained followed by evaporation to dryness and reconstitution with 80 μL MeOH/0.1% NH_3_·H_2_O in H_2_O (80:20, v/v) for injection of 10 μL supernatant into HPLC-MS/MS system for analyses of emodin. Furthermore, the concentrations of total emodin (emodin and emodin glucuronides) were determined after hydrolysis that 100 μL plasma or liver homogenate was mixed with 50 μL β-glucuronidase (2000 units/mL in 0.5 M acetate buffer, pH 5) and 50 μL ascorbic acid (100 mg/ml) followed by incubation at 37°C for 2 h. The hydrolysis reaction was terminated by adding 100 μL ice-cold MeOH containing 1 μg/ml Daidzein (IS) and the concentrations of emodin glucuronides were gained *via* subtracting the concentration of free form emodin from the total concentrations of emodin.

#### 2.4.4 Determination of bile acids in rat liver

Fifteen bile acids, including cholic acid (CA), chenodeoxycholic acid (CDCA), deoxycholic acid (DCA), lithocholic acid (LCA), ursodeoxycholic acid (UDCA), and the taurine and glycine conjugates of these bile acids in the liver homogenate were determined as described in previous reports (Huang et al., 2011; Li et al., 2022). In brief, liver homogenate (200 μL) was spiked with 10 μL internal standard solution (daidzein, 5 μg/ml) and 790 μL ACN comprising 5% ammonium solution (95/5, v/v), and the mixture was centrifuged at 20,000 g for 10 min to obtain 800 μL supernatant for vacuum evaporation. The obtained residue was then reconstituted with 80 μL MeOH/H_2_O (50:50, v/v), centrifuged at 20,000 g for 10 min and 10 μL of the supernatant was subjected to HPLC–MS/MS for analyses of bile acids. All the fifteen bile acids were separated on a Welch Materials Ultimate HPLC XB-C18 column (2.1 × 100 mm, 3 μm) at a flow rate of 0.3 ml/min. The mobile phase consisted of an aqueous solution of 7.5 mM ammonium formate adjusted to pH = 7.0 with NaOH (A) and MeOH (B). The gradient elution started from 40% solvent B to 90% solvent B within 22 min. The sample was ionized *via* an ESI source in the negative mode and monitored in the multiple reaction monitoring (MRM) mode. The gas temperature was set at 350°C and the nebulizer gas was 12 psi.

#### 2.4.5 Investigation on protein expression of bile acid homeostasis- and apoptosis-related proteins in rat liver

To better understand the molecular regulation, the protein expression of bile acid homeostasis related proteins including key bile acid synthesis enzymes (CYP7A1, CYP27A1) and major bile acid efflux transporters (BSEP, MRP2, MRP3, MRP4) as well as the apoptosis-related proteins (BAX, BCL-2, Cleaved CASPASE-9 and Cleaved CASPASE-3) in the rat liver were examined by western blot assay. The liver samples were treated with RIPA lysis buffer containing protease inhibitors cocktail to extract the total protein and the concentrations of protein were analysed by BCA protein assay kit according to the manufacturer’s instructions. About 50 μg protein was separated by 7.5% or 12% sodium dodecyl sulfate polyacrylamide gel electrophoresis. Subsequently, the protein was transferred to PVDF blotting membranes for 2 h *via* the wet transfer method. Then, the membranes were blocked for 1 h with 5% BSA in 0.1% Tris-Buffered Saline Tween-20 (TBST). After that, the membranes were incubated overnight at 4°C with the primary antibodies including CYP7A1 (1:200), CYP27A1 (1:200), BSEP (1:200), MRP2 (1:1000), MRP3 (1:1000), MRP4 (1:1000), BCL-2 (1:200), BAX (1:200), Cleaved CASPASE-3 (1:1000), Cleaved CASPASE-9 (1:1000) and β-ACTIN (Abcam, 1:5000). Subsequently, the membranes were further incubated with the corresponding secondary antibodies (1:5000) for 1 h at room temperature and the protein bands were then visualized *via* the enhanced chemiluminescence detection system with β-ACTIN serving as the housekeeping protein.

### 2.5 Data analyses

The total bile acids (TBA) levels were calculated as the sum of levels for CA, CDCA, DCA, UDCA, LCA, taurine and glycine conjugated bile acids. The amidated bile acids levels referred to the sum of levels for all the taurine and glycine conjugated bile acids, and the unconjugated bile acids levels were that of CA, CDCA, DCA, UDCA, and LCA. Total primary bile acids levels were the sum of levels for CA, CDCA, UDCA, and their taurine and glycine conjugated bile acids. Total secondary bile acids levels were the sum of levels for DCA and LCA and their corresponding taurine and glycine conjugated bile acids. Total CA levels were the sum of levels for CA, taurine and glycine conjugated CA, and the total CDCA, DCA, UDCA and LCA were calculated with the same method of total CA.

All the data were presented as mean ± standard deviation (SD) and subjected to the two-way analysis of variance (ANOVA) followed by the Tukey post hoc test or students’ t-test. *p* < 0.05 was considered to be statistically significant.

## 3 Results

### 3.1 Increased systemic and hepatic exposure of total emodin after combination treatment with emodin and TSG in both normal and LPS-mediated inflammatory stress model rats

**FIGURE 1:**
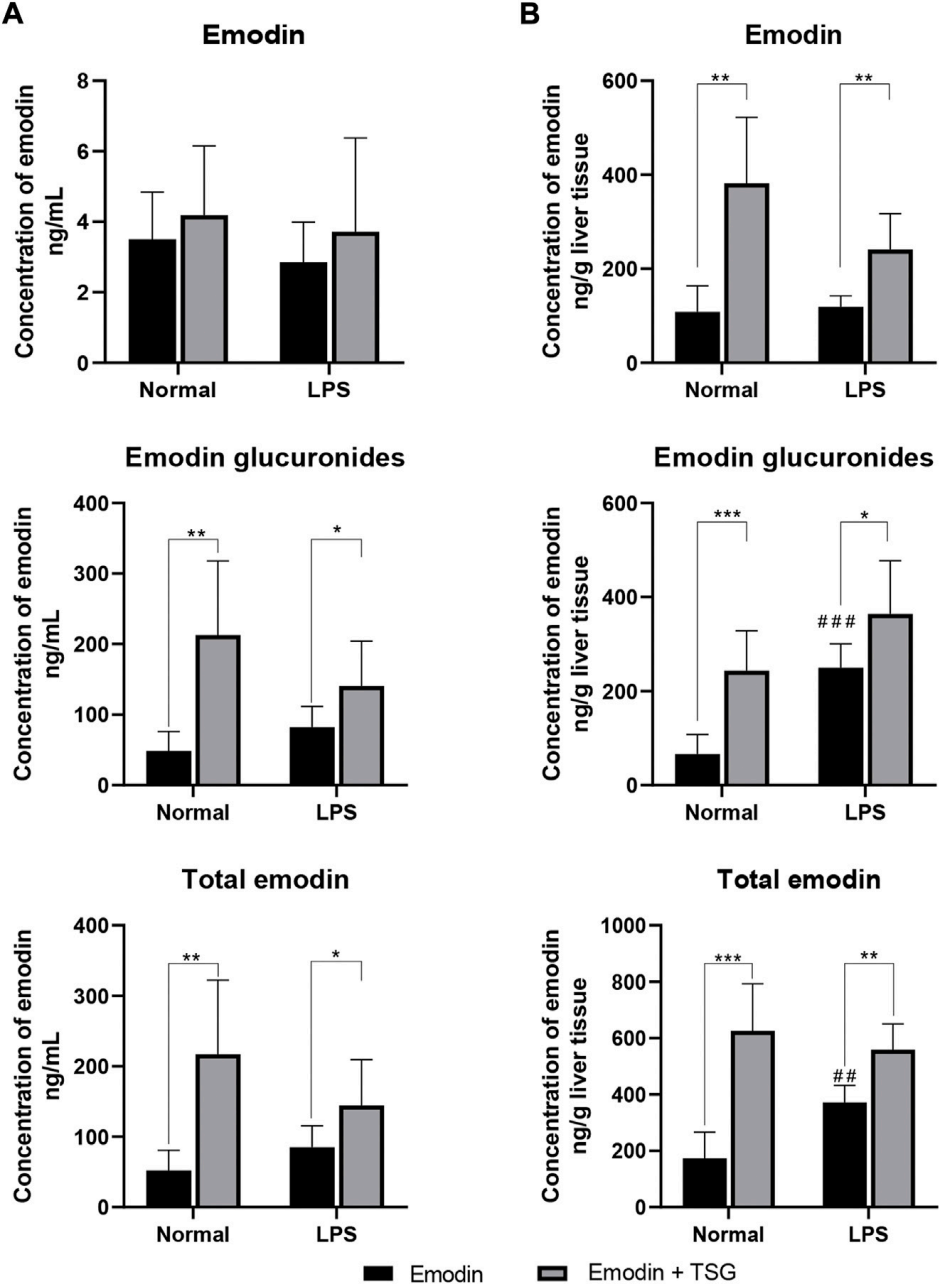
Concentrations of emodin, emodin glucuronides, and total emodin (emodin + emodin glucuronides) in plasma **(A)** and liver **(B)** after treatment with emodin or its combination with TSG in the normal or LPS-mediated inflammatory stress model rats. The data are expressed as the mean ± SD (*n* = 6). *p < 0.05, **p < 0.01, ***p < 0.001 by Student’s t-test compared between the Emodin and Emodin + TSG groups, ##*p* < 0.01, ###*p* < 0.001 by Student’s t-test compared with Emodin group in normal rats.

As displayed in Figure 1A, although the parent form of emodin in the plasma showed no significant difference between the emodin-treated and its combination treatment with TSG groups in either the normal or LPS-mediated inflammatory stress model rats, the plasma concentrations of emodin glucuronides and total emodin (emodin + emodin glucuronides) were significantly increased after combination treatment with emodin and TSG in both the normal and LPS-mediated inflammatory stress model rats. In addition, after emodin administrations, the hepatic concentrations of emodin glucuronides and total emodin (emodin + emodin glucuronides) in the LPS-mediated inflammatory stress model rats were significantly higher than those in the normal rats (Figure 1B). Furthermore, it was found that the hepatic concentrations of emodin and its glucuronides as well as total emodin (emodin + emodin glucuronides) after the combination treatment with emodin and TSG in both the normal and LPS-mediated inflammatory stress model rats were all significantly elevated compared to that from the emodin individual treatment group as shown in Figure 1B.

### 3.2 Impact of the LPS treatment on the proinflammatory cytokines, liver injury indexes, hepatic bile acid accumulation, and expressions of bile acid homeostasis and apoptosis related proteins

**FIGURE 2:**
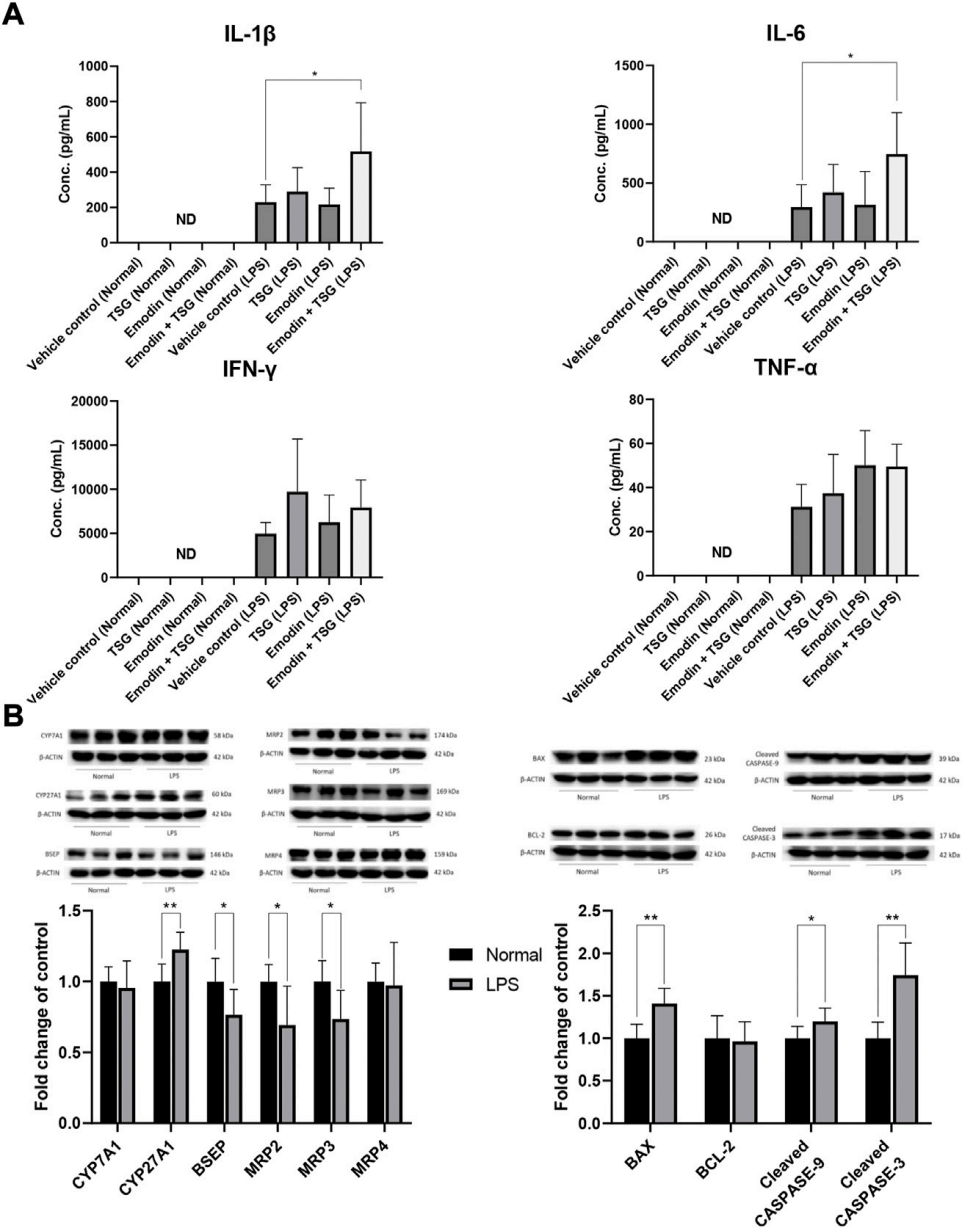
Plasma levels of the inflammatory cytokines after oral administrations of vehicle control, TSG, emodin and their combination to normal rats (Normal) and LPS-mediated inflammatory stress model rats (LPS), *p < 0.05 by two-way ANOVA followed by the Tukey post hoc test, ND, not detectable **(A)**; protein expression of bile acid synthesis enzymes (CYP7A1, CYP27A1), efflux transporters (BSEP, MRP2, MRP3, MRP4) and apoptosis-related proteins (BAX, BCL-2, Cleaved CASPASE-9, Cleaved CASPASE-3) in the liver after oral administration with vehicle control to normal or LPS-mediated inflammatory stress model rats, *p < 0.05, **p < 0.01 by Student t-test **(B)**. Data are represented as mean ± SD (*n* = 6).

**FIGURE 3:**
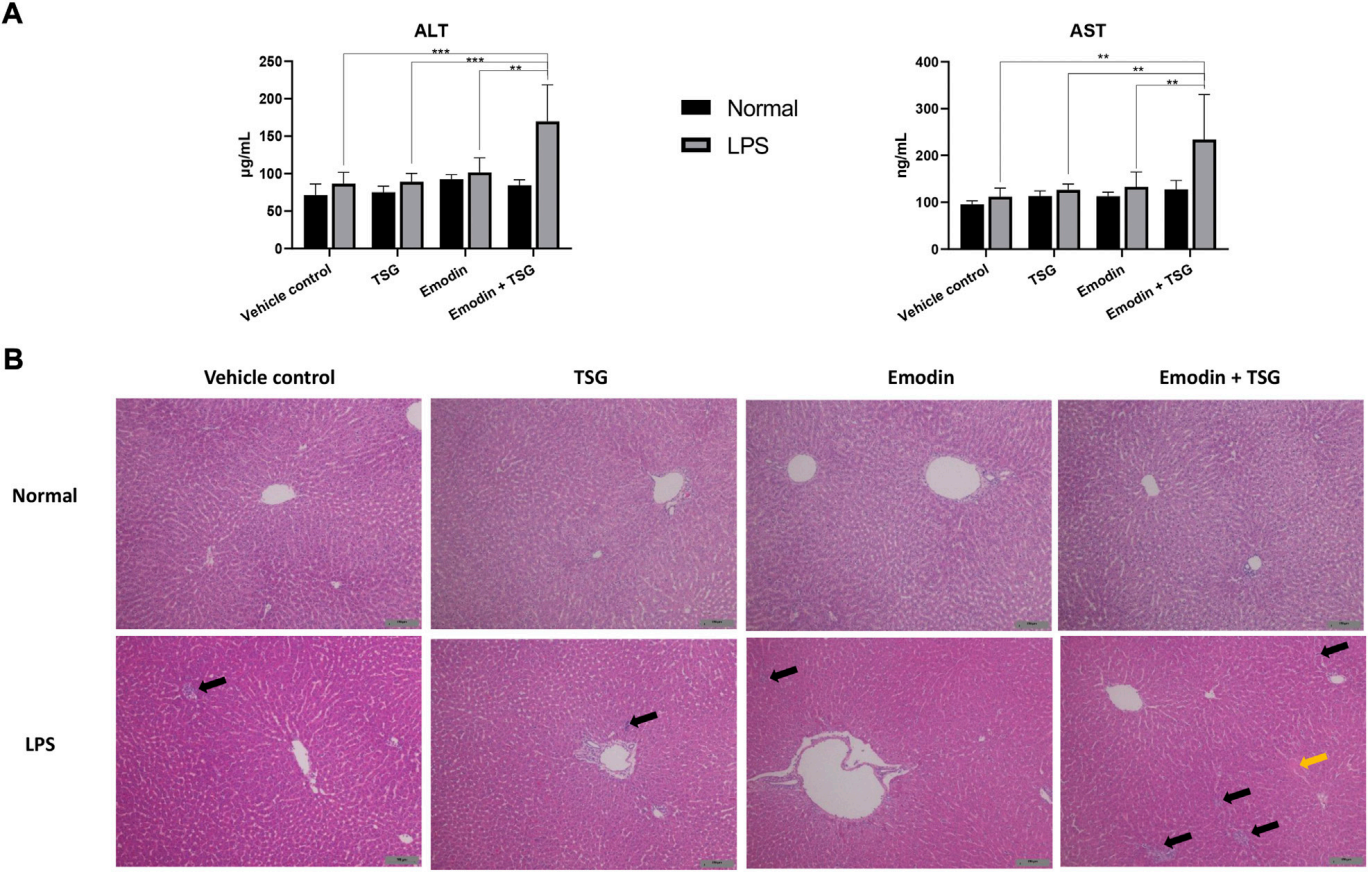
Plasma levels of ALT and AST **(A)** and histology changes in the liver **(B)**, H&E staining, ×100 magnification) after treatment with emodin, TSG, or their combination in normal and LPS-mediated inflammatory stress model rats. The data are expressed as mean ± SD (*n* = 6). **p < 0.01, ***p < 0.001 by two-way ANOVA followed by the Tukey post hoc test. The solid black arrows indicated inflammatory cell infiltration, and the solid yellow arrow pointed to cell swelling. The scale bar represents 100 μm.

**FIGURE 4:**
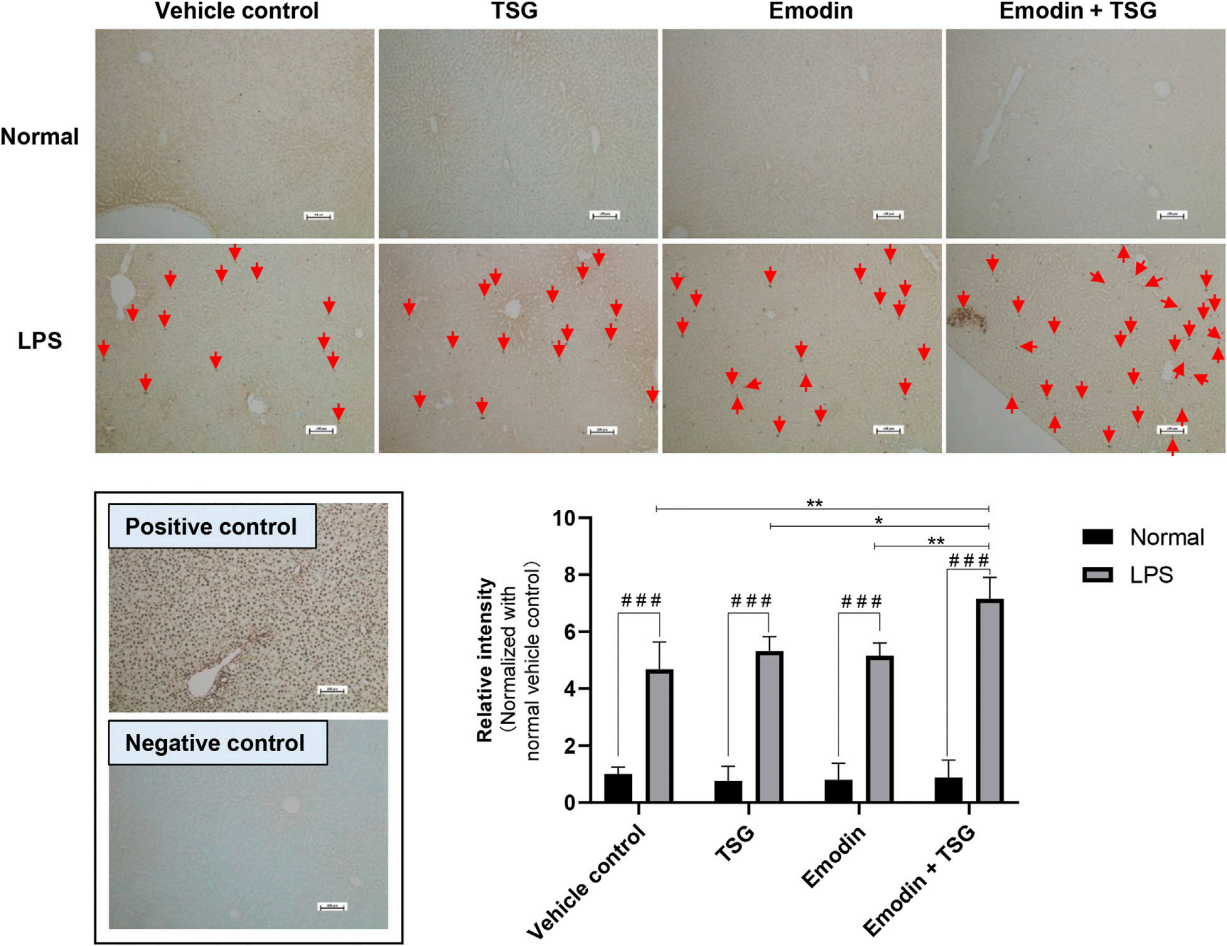
Representative images and quantitative analysis of apoptotic hepatocytes in the rat livers after treatment with emodin, TSG, or their combination in normal and LPS-mediated inflammatory stress model rats (TUNEL staining, ×100 magnification). Positive control: rat liver section treated with DNase I. Negative control: TdT enzyme was substituted with water. Apoptotic cells (Red Arrow) were labeled with TdT and signals were developed using DAB. Sections were counterstained with Methyl Green. The data are expressed as the mean ± SD (*n* = 6). ^# # #^ *p* < 0.001 by Student’s t-test, *p < 0.05, **p < 0.01 by two-way ANOVA followed by the Tukey post hoc test. The scale bar represents 100 μm.

**FIGURE 5:**
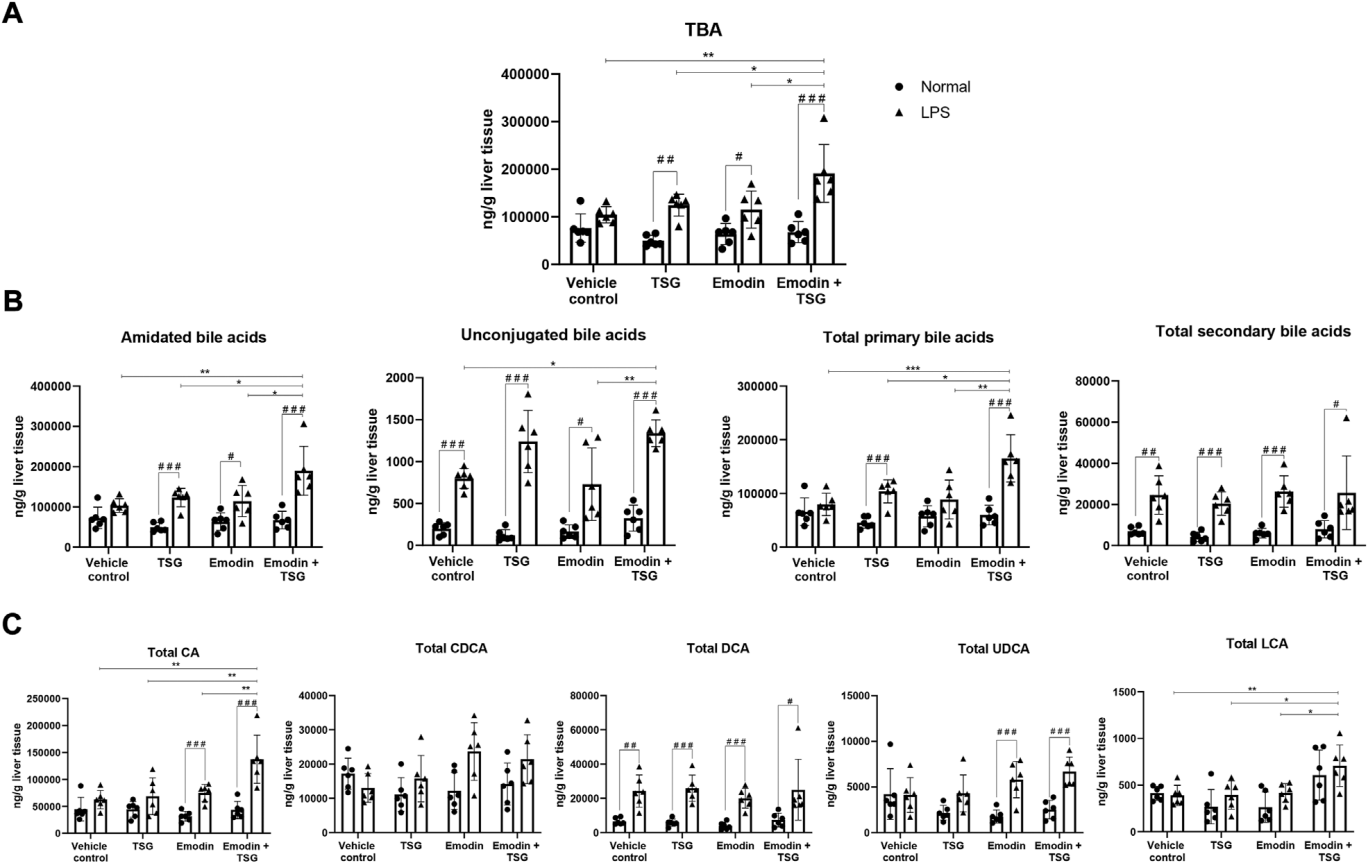
Hepatic concentrations of TBA **(A)**, amidated bile acids, unconjugated bile acids, total primary bile acids, and total secondary bile acids **(B)** and total CA, total CDCA, total DCA, total UDCA, and total LCA **(C)** after oral administration of vehicle control, TSG, emodin or their combination to normal and LPS-mediated inflammatory stress model rats. The data are expressed as mean ± SD (*n* = 6). ^#^ *p* < 0.05, ^*# #*^ *p* < 0.01, ^# # #^ *p* < 0.001 by Student’s t-test, *p < 0.05, **p < 0.01, ***p < 0.001 by two-way ANOVA followed by the Tukey post hoc test.

It was noted that plasma levels for the studied inflammatory mediators, including IL-1β, IL-6, TNF-α, and IFN-γ were significantly increased in the LPS-mediated inflammatory stress model rat groups in comparison with that of the normal groups (no detectable inflammatory cytokines) as shown in Figure 2A, indicating the successful establishment of the LPS-mediated inflammatory stress rat model. In addition, compared to the normal rats treated with vehicle control, the protein level of bile acid synthesis enzyme CYP27A1 was significantly increased in the LPS-mediated inflammatory stress model rats treated with vehicle control. In the meantime, the protein expressions of bile acid efflux transporters, BSEP, MRP2 and MRP3 were significantly decreased with no change observed in that of MRP4 in the LPS-mediated inflammatory stress model rats compared to the normal rats (Figure 2B). Moreover, LPS was also found to significantly up-regulate the protein expressions of BAX, Cleaved CASPASE-9 and Cleaved CASPASE-3 (Figure 2B). The original images for Figure 2B are provided in the Supplementary Material. Although no significant difference was observed in the plasma levels of ALT and AST between the normal rats and LPS-mediated inflammatory stress model rats after treatment with vehicle control as displayed in Figure 3A, slight inflammatory cell infiltration was observed in rat liver administered with vehicle control (Figure 3B). The images and quantitative analysis of the apoptotic hepatocytes identified by TUNEL analyses are displayed in Figure 4 with dark brown signals indicating apoptotic hepatocytes. It was noted that the liver samples of positive control for the TUNEL staining assay contained strong immunoreactive signals, whereas only background staining signal appeared in that of the negative control. It was also observed that the LPS treatment itself could also induce scattered apoptotic hepatocytes after bolus dosing with vehicle control. As shown in Figure 5, after treating the rats with vehicle control, the unconjugated bile acids/total secondary bile acids/total DCA in the liver were significantly increased in the LPS-mediated inflammatory stress model rats with no significant change observed for the TBA/amidated bile acids/total primary bile acids/total CA/total CDCA/total UDCA/total LCA compared to that from the normal rats.

### 3.3 Liver injury induced by the bolus combination treatment with emodin and TSG in LPS-mediated inflammatory stress model rats

#### 3.3.1 Increased inflammatory, liver injury indexes and liver histopathological changes

Among the LPS-mediated inflammatory stress model rats receiving different treatments, the results indicated that rats with the bolus combination treatment with emodin and TSG demonstrated significant elevation on the plasma levels of IL-1β and IL-6 in comparison to that from the vehicle control group (Figure 2A). As shown in Figure 3A, neither the individual treatment with emodin or TSG nor their combination treatment altered the plasma levels of ALT and AST among the normal rats receiving the different treatments. However, among the LPS-mediated inflammatory stress model rats, approximately 96% elevation of ALT and 109% of AST in the plasma were only induced by the bolus combination treatment with emodin and TSG compared to that from the vehicle control group (Figure 3A). As displayed in Figure 3B, slight inflammatory cell infiltration was noted in rat livers administered with TSG or emodin in the LPS-mediated inflammatory stress model rats, whereas severe inflammatory cell infiltration and cell swelling were displayed after bolus combination treatment with emodin and TSG in the LPS-mediated inflammatory stress model rats. In addition, as shown in Figure 4, the number of apoptotic hepatocytes in the LPS-mediated inflammatory stress model rats treated with emodin, TSG or their combination were significantly increased in comparison to that from the normal rats. Furthermore, although neither emodin/TSG nor their combination treatment showed a significant difference from that in the normal vehicle control group in the normal rats, the significantly enhanced number of apoptotic hepatocytes was observed after the bolus combination treatment with emodin and TSG in LPS-mediated inflammatory stress model rats (Figure 4).

#### 3.3.2 Bile acid homeostasis disruption

After receiving TSG, emodin or their bolus combination treatments, the hepatic levels of TBA/amidated bile acids/unconjugated bile acids/total primary/secondary bile acids/total CA/total DCA/total UDCA in the LPS-mediated inflammatory stress model rats were significantly increased than that from the normal rats as displayed in Figure 5. Among the normal rats, neither TSG/emodin nor their combination affected the disposition of TBA/amidated bile acid/unconjugated bile acids/total primary bile acids/total secondary bile acids/total CA/total CDCA/total DCA/total UDCA/total LCA in the liver. However, in the LPS-mediated inflammatory stress model rats, compared to the vehicle control or individual treatment groups, the bolus combination treatment with emodin and TSG significantly increased the hepatic concentrations of TBA (Figure 5A), amidated bile acids/unconjugated bile acids/total primary bile acids (Figure 5B) in the LPS-mediated inflammatory stress model rats. Furthermore, compared with the vehicle control or the individual treatment groups, the bolus combination treatment with emodin and TSG significantly increased the hepatic levels of total CA and total LCA with no change observed on total CDCA/total DCA/total UDCA in the LPS-mediated inflammatory stress model rats as shown in Figure 5C.

#### 3.3.3 Alterations on protein expression levels of bile acid homeostasis- and apoptosis-related proteins

**FIGURE 6:**
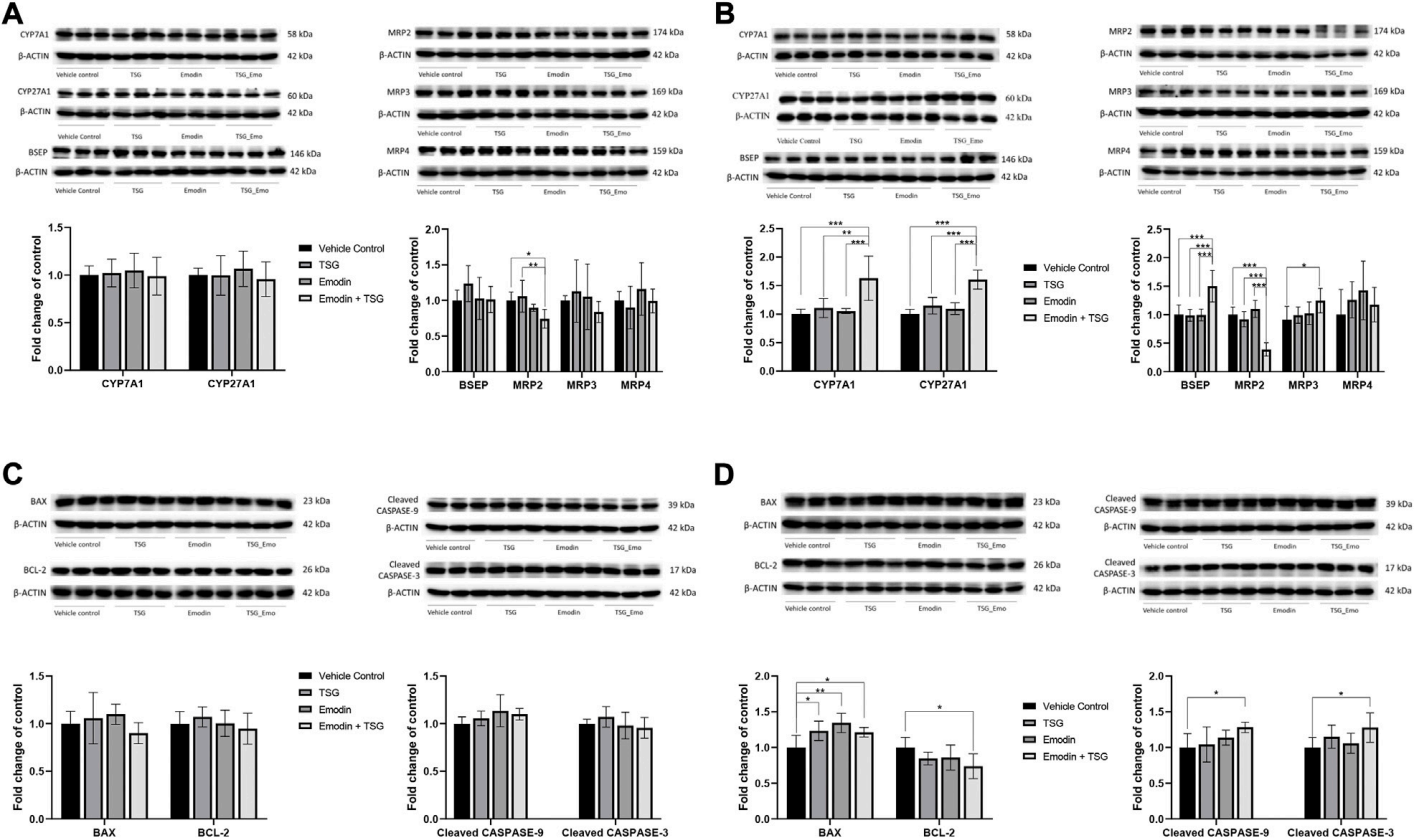
Protein expression of bile acid synthesis enzymes (CYP7A1 and CYP27A1), bile acid efflux transporters (BSEP, MRP2, MRP3, and MRP4) and apoptosis-related proteins (BAX, BCL-2, Cleaved CASPASE-3, and Cleaved CASPASE-9) in the liver after oral administration with emodin, TSG or their combination to normal rats **(A,C)** or LPS-mediated inflammatory stress model rats **(B,D)**. The data are expressed as mean ± SD (*n* = 6). *p < 0.05, **p < 0.01, ***p < 0.001 by two-way ANOVA followed by the Tukey post hoc test.

The hepatic protein expression levels of key bile acid synthesis enzymes (CYP7A1, CYP27A1) and major bile acid efflux transporters including BSEP, MRP2, MRP3, and MRP4 were examined and shown in Figure 6. In the normal rats, except for MRP2, which was significantly inhibited by the bolus combination treatment with emodin and TSG compared with the vehicle control or TSG groups, the other proteins were not affected by the treatment with emodin, TSG, or their combination (Figure 6A). Similarly, as shown in Figure 6B, the protein expression level of MRP2 was also significantly decreased by the bolus combination treatment with emodin and TSG in the LPS-mediated inflammatory stress model rats. At the same time, that of BSEP and MRP3 was markedly elevated in the TSG and emodin combination treatment group in comparison to the vehicle control or individual treatment groups and no change was observed in the expression of MRP4. In addition to these bile acid transporters, also indicated in Figure 6B, the protein expressions of CYP7A1 and CYP27A1, the key bile acid synthesis enzymes, were also significantly increased by the bolus combination treatment with emodin and TSG in the LPS-mediated inflammatory stress model rats.

Consistently with the TUNEL staining results, both the individual treatment with TSG or emodin and their combination treatment showed no impact on these four proteins in the normal rats (Figure 6C). However, in the LPS-mediated inflammatory stress model rats (Figure 6D), the protein expression of BAX, the pro-apoptotic protein, was significantly up-regulated by both the individual and combination treatment with emodin or TSG. In the meantime, that of BCL-2, the anti-apoptotic protein, was significantly decreased after the combination treatment with emodin and TSG compared to the vehicle control group. Similar to the results of BCL-2, it was found that only the bolus combination treatment with emodin and TSG, instead of the individual treatment with emodin or TSG, significantly up-regulated the protein expression levels of Cleaved CASPASE-9 and Cleaved CASPASE-3 (Figure 6D). The original images for Figure 6 are provided in the Supplementary Material.

## 4 Discussions

Although previous studies have investigated the liver injury induced by PMR or its major components in normal rats after either long-term exposure/overdose or in the LPS-mediated inflammatory stress model rats (Li et al., 2020), the preparations and dose of PMR varied among different studies and the doses of the PMR pure components adopted in these studies were usually much higher than their clinically relevant doses in PMR (Tu et al., 2015; Meng et al., 2017; Zhang et al., 2020). Moreover, as demonstrated in our previous report (Li et al., 2022), emodin was identified as the most toxic component and its enhanced hepatotoxicity with TSG cotreatment could contribute to PMR-induced liver injury after long-term administration. Therefore, in the current study, the idiosyncratic liver injury caused by the bolus combination treatment with emodin and TSG in the LPS-mediated inflammatory stress model rats was explored for the first time.

The idiosyncratic liver injury has been investigated in several models, including the Inflammatory stress model, Uetrecht-Pohl model and Subclinical Mitochcondriopathy model, among which the Inflammatory stress model triggered by LPS is the most commonly used one due to its convenience for use and easy accessibility (McGill and Jaeschke, 2019). LPS could activate the macrophages, thus leading to the inflammatory cells stimulation and resulted expression and release of many proinflammatory cytokines such as IL-1β, IL-6, TNF-α and IFN-γ, which may increase the liver cell sensitivity to xenobiotics and liver injury in aggravated conditions (Ganey et al., 2004). The modest inflammation triggered by a non-toxic dose of bacterial LPS in the animals could decrease the threshold for xenobiotic hepatotoxicity (Rotha et al., 1997; Ganey et al., 2004). It was noted that the plasma levels of inflammatory cytokines in our current study were different from the previous reports for the same type of rat with same LPS treatments (Gao et al., 2016b; Meng et al., 2017). Such discrepancy may be due to the different ELISA kits adopted in our study (Abcam, Cambridge, MA, United States) from theirs (Cloud-Clone Corp. Houston, TX, United States). The plasma levels of IL-1β, IL-6, TNF-α and IFN-γ in the normal rats detected in our study were lower than the detection limits of our ELISA kits, which are 54.69 pg/ml, 125 pg/ml, 18.75 pg/ml and 7.0 pg/ml, respectively, while the detection limits of those ELISA kits adopted by Meng et al. and Gao et al. are 15.6 pg/ml for IL-1β, 7.8 pg/ml for IL-6, 15.6 pg/ml for TNF-α and 15.6 pg/ml for IFN- γ. In our current study, the liver injury induced by the bolus combination treatment with emodin and TSG in LPS-mediated inflammatory stress model rats could result from the decreased threshold for xenobiotic hepatotoxicity due to the inflammatory responses stimulated by LPS. Such phenomenon was also observed for other agents that can induce idiosyncratic liver injury in clinical practice, including western drugs such as chlorpromazine (Buchweitz et al., 2002), diclofenac (Deng et al., 2006) and halothane (Dugan et al., 2010), ranitidine (Shaw et al., 2007), trovafloxacin (Waring et al., 2006) and herbal medicines such as PMR (Gao et al., 2016b), Dictamni Cortex (Ganey et al., 2004; Roth and Ganey, 2011; Wei et al., 2019) and Tripterygium wilfordii (Huang et al., 2008).

The doses of emodin (5 mg/kg) and TSG (165 mg/kg) adopted in our current study were based on 1) the maximum hepatotoxic dose of PMR (200 g/person/day) in clinical practice (Tu et al., 2019a), which is equivalent to 20.6 g crude material/kg in rats, and 2) the contents of total emodin (emodin and EMG) and TSG in PMR concentrated granules (total emodin: 24 mg/20 g granules or 100 crude material; TSG: 800 mg/20 g granules or 100 crude material) reported in our previous study (Li et al., 2021). The significant elevation of hepatic or systemic exposure of emodin was observed in the normal and LPS-mediated inflammatory stress model rats after a bolus combination treatment with emodin and TSG, which was consistent with our previous report that TSG could significantly increase the hepatic or systemic exposure of emodin in the SCRH and normal rats after consecutively 21-days treatment (Li et al., 2022). Moreover, consistently with the previous findings with TSG dose up to 500 mg/kg in the LPS-mediated inflammatory stress model rats (Zhang et al., 2020), we also did not observe any liver injury with TSG treatment at 165 mg/kg in the LPS-mediated inflammatory stress model rats in the current study. Therefore, the resulted liver injury of the bolus combination treatment with emodin and TSG in the LPS-mediated inflammatory stress model rats was considered to be mainly due to the significant elevation of the hepatic exposure of emodin induced by TSG. Since transporters including MRP2, MRP3, and MRP4 were involved in the excretion of emodin glucuronides from the hepatocyte into the blood or bile (Liu et al., 2012), inhibition of the protein expression of MRP2 could be a part of the reason for the increased hepatic exposure of emodin glucuronides after the bolus combination treatment with emodin and TSG in both normal and LPS-treated rats. In addition, the glucuronides of emodin were found to be significantly increased after treatment with emodin in the LPS-mediated inflammatory stress model rats compared to the normal rats. The plausible explanation could be the inhibited expression of MRP2 after LPS treatment, which was consistent with the reported literature (Aitken et al., 2006). However, such elevation was not observed after bolus combination treatment with emodin and TSG between the normal and LPS-mediated inflammatory stress model rats, which might be due to the increased protein expression of MRP3 located at the basolateral side of the hepatocyte.

The hepatic bile acids accumulation was reported to exert cytotoxic effects *via* impairing cell membrane integrity (Schadt et al., 2016). In addition, the previous study has also suggested the bile acid homeostasis disruption as an early pathogenesis event of DILI (Yamazaki et al., 2013). Bile acid homeostasis is closely correlated with bile acid transport and biosynthesis. According to the previous findings in both rats and humans, exposure to LPS could decrease hepatic mRNA levels of the bile acid transporters such as *NTCP/Ntcp* (Cherrington et al., 2004), *BSEP/Bsep* (Vos et al., 1998; Hartmann et al., 2002; Elferink et al., 2004), OATPs/*Oatps* (Hartmann et al., 2002; Cherrington et al., 2004), *MRP2/Mrp2* (Trauner et al., 1997; Vos et al., 1998; Hartmann et al., 2002; Elferink et al., 2004) and *MRP3/Mrp3* (Hartmann et al., 2002) due to the elevation of inflammatory cytokines (Hartmann et al., 2002; Lee and Miller, 2003). Our study has also demonstrated that LPS could significantly increase the protein expression level of CYP27A1 and inhibit that of BSEP, MRP2 and MRP3. Such regulation could be used to explain the hepatic accumulation of the unconjugated bile acids and the total secondary bile acids after the vehicle control administration in the LPS-mediated inflammatory stress model rats compared to the normal rats in our current study.

The significant inhibition on the protein level of MRP2 caused by the bolus combination treatment with emodin and TSG was considered to contribute to the hepatic accumulation of bile acids in the LPS-mediated inflammatory stress model rats. Contrary to the inhibition of MRP2, the significant up-regulation of BSEP and MRP3 was regarded as an adaptive response to release the hepatic accumulation of bile acids (Zollner et al., 2006), which was also consistent with the significant up-regulation of protein expression levels of BSEP, MRP2, MRP3 and MRP4 after treating the normal rats with emodin and TSG for consecutively for 21 days (Li et al., 2022). As indicated before, the bile acids are mainly synthesized *via* the classic pathway and alternative pathway, which are initiated by the rate-limiting enzyme CYP7A1 and CYP27A1, respectively (Chiang, 2017). The significant up-regulation of bile acid synthesis enzymes, CYP7A1 and CYP27A1, was another important reason for the hepatic accumulation of bile acid induced by the bolus combination treatment with emodin and TSG in the LPS-mediated inflammatory stress model rats after bolus dose administration. In addition, such elevation of CYP7A1 and CYP27A1 was also consistent with the increase in the hepatic accumulation of total CA or total CDCA, the products of these two enzymes (Molinaro et al., 2018). However, the classic pathway involving CYP7A1 contributes to about 80% TBA production in the humans and about 50% in the rodents under physiological conditions, respectively (Chiang and Ferrell, 2018). The contribution of the elevated expression level of CYP27A1 to the PMR-induced idiosyncratic liver injury in humans needs further exploration. In addition to the inhibition of the protein expression of the bile acids efflux transporters and induction of bile acids synthesis enzymes, the significant hepatic accumulation of total LCA, the most toxic bile acid (Ashby et al., 2018), was considered to be another important factor for the idiosyncratic liver injury induced by the bolus combination treatment with emodin and TSG.

Besides the disruption of bile acids synthesis and transport, apoptosis also plays an important role in the physiological process and achievement of tissue homeostasis. The induction of hepatocytes apoptosis was suggested to involved in the pathogenesis of some liver diseases (Guicciardi and Gores, 2005). Both the bile acid (Perez and Briz, 2009) and emodin (Dong et al., 2018; Lin et al., 2019; Yang et al., 2019; Zheng et al., 2019) were reported to induce apoptosis. Our current study for the first time demonstrated that the bolus combination treatment with emodin and TSG instead of their individual treatment induced liver apoptosis after a bolus treatment in the LPS-mediated inflammatory stress model rats, which was consistent with the results from the normal rats after consecutively 21-days treatment (Li et al., 2022). Both the hepatic accumulation of emodin and bile acid may contribute to the occurrence of apoptosis after the bolus combination treatment with emodin and TSG in the LPS-mediated inflammatory stress model rats.

Herb-induced liver injury is the result of the interaction between the host and herb, in which the herbal components serve as the major source for hepatotoxicity. The previous reports extensively explored the risk factors from the host perspective for PMR hepatotoxicity (Ma et al., 2014; Li et al., 2017; Zhang et al., 2018; Li et al., 2019), while our previous report (Li et al., 2022) and the current investigation focus on the herbal components perspective and demonstrated the contribution of emodin together with TSG to PMR hepatotoxicity. In clinical practice, the PMR-induced liver injury could be attenuated or recovered after discontinuing PMR products and conservative care (Lei et al., 2015). Our studies suggested that monitoring the systemic exposure levels of emodin would be an effective way to adjust the PMR doses so as to prevent the occurrence of PMR-induced hepatotoxicity in clinical practice. In addition, since the disruption of bile acids homeostasis was involved in the PMR hepatotoxicity, therefore, attenuating the cholestasis would be another way for the treatment of PMR-induced hepatotoxicity. Moreover, according to the Chinese Pharmacopeia (Chinese Pharmacopoeia Commission, 2020) and Hong Kong Materia Medical Standards (Hong Kong Chinese Materia Medica Standards, 2008), there are only lower limit requirements for the contents of emodin and TSG in the crude PMR material. Based on our identified hepatotoxicity induced by emodin (Li et al., 2022) and its combination use with TSG, setting upper content limits for these two components in the crude PMR material would be another effective way to ensure the safe use of PMR in clinical practice.

## 5 Conclusion

Bolus combination treatment with emodin and TSG could induce idiosyncratic liver injury due to the increased hepatic exposure of emodin, hepatic accumulation of bile acids and apoptosis, which was considered to contribute to PMR-induced idiosyncratic liver injury.

## Data Availability

The original contributions presented in the study are included in the article/Supplementary Material, further inquiries can be directed to the corresponding author.
